# Exploring challenges of RCTs in the emergency and critical care setting

**DOI:** 10.1186/1745-6215-16-S2-O23

**Published:** 2015-11-16

**Authors:** Paul Mouncey, Sarah Power, David Harrison, Sheila Harvey, Kathryn Rowan

**Affiliations:** 1 ICNARC Clinical Trials Unit, London, UK

## Introduction

Approximately 50% of randomised controlled trials (RCTs) fail to recruit to time and target. RCTs in the emergency and critical care settings pose additional challenges including time of presentation of potentially eligible patients and the time required to formally recruit patients.

## Objectives

To describe the screening and recruitment patterns in RCTs in an emergency/critical care setting in the NHS and to explore the impact that time taken to consent patients may have on the delivery of an early intervention.

## Methods

We recently completed two large multicentre RCTs in the emergency and critical care setting. Both evaluated delivery of an early intervention - early, goal-directed therapy (a resuscitation protocol) (n=1260 patients; 56 sites) and early nutritional support (n=2400 patients; 33 sites). As well as collecting data for the evaluation, we also collected data around screening, recruitment and timing.

## Results

In both trials, due to the setting, recruitment rates were lower - with eligible patients missed - at nights and at weekends with an absence of study “delegated” staff available often cited as the reason. In both trials, 81% of patients were recruited Monday to Friday 08:00 to 19:59. In the trial on resuscitation, time taken to formally recruit patients impacted on the early nature of the intervention (meeting inclusion criteria to randomisation was on average 1.1 hours).

## Conclusions

Until research infrastructure can be delivered 24/7, RCTs in emergency and critical care settings will struggle to deliver to time and target. The use of deferred consenting procedures for time-sensitive interventions warrants further debate.

